# Cephalic Musculature of the Pacman Catfish *Lophiosilurus alexandri* Steindachner, 1876 (Siluriformes, Pseudopimelodidae)

**DOI:** 10.1002/jmor.70056

**Published:** 2025-05-24

**Authors:** Rafael da Silva Marques, Isabela Ohara, Oscar Akio Shibatta

**Affiliations:** ^1^ Programa de Pós‐Graduação em Ciências Biológicas, Departamento de Biologia Animal e Vegetal, Centro de Ciências Biológicas Universidade Estadual de Londrina Londrina Brazil; ^2^ Museu de Zoologia, Departamento de Biologia Animal e Vegetal, Centro de Ciências Biológicas Universidade Estadual de Londrina Londrina Brazil

**Keywords:** anatomy, catfish, freshwater, São Francisco River basin, systematics

## Abstract

The cephalic musculature of the Pacman catfish *Lophiosilurus alexandri* (*L. alexandri*) is described and compared with *Pimelodus maculatus, Pimelodus microstoma*, *Pseudopimelodus mangurus* (*P. mangurus*), *Batrochoglanis labrosus* (*B. labrosus*), and *Lophiosilurus fowleri* (*L. fowleri*). Besides the distinguished Pacman catfish head shape, which is strongly depressed, broad, and with a large mouth, we hypothesize that the gross morphology of the musculature is related to the phylogenetic background. A phylogenetic analysis of selected characters evidenced three putative synapomorphies for the family Pseudopimelodidae, three for the subfamily Batrochoglaninae, three for the genus *Lophiosilurus*, two autapomorphies for *L. alexandri*, one for *L. fowleri*, one for *B. labrosus*, and five for *P. mangurus*. The absence of the *retractor tentaculi* is interpreted as a putative synapomorphy of Pseudopimelodidae and Pimelodidae. The rounded *adductor mandibulae* emerge as the predominantly voluminous musculature in *L. alexandri* and other Pseudopimelodidae, a conspicuous synapomorphy of the family. Profound differences were observed when comparing the cephalic musculatures of *L. alexandri* with *Lophius piscatorius* and *Chaca bankanensis*, which are unrelated species with similar body morphology and ambush behavior. The morphology of cephalic musculature highlights the plasticity of the musculature function and the closer relationship with the phylogenetic history of species and lineages.

## Graphical abstract

The head of the Pacman catfish (*Lophiosilurus alexandri* [*L. alexandri*]) is strongly depressed, broad, and with a large mouth, roughly resembling those of *Lophius piscatorius* and *Chaca bankanensis*. Also, they are bottom dwellers and have ambush behavior for fish predation. However, profound differences were observed when comparing the cephalic musculatures of *L. alexandri* with those species. In contrast, the phylogenetic analysis evidence muscular phylogenetic relationship within the family. Therefore, the morphological plasticity of the musculature was evident, as was its relationship with the phylogenetic history of species and lineages.

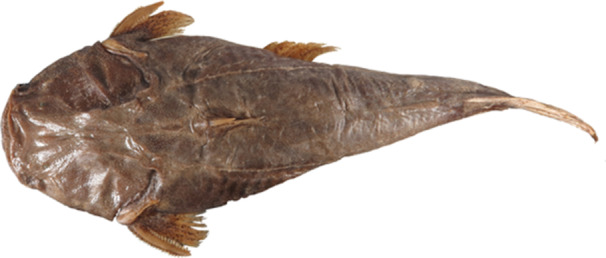

## 1 Introduction

Siluriformes, that is, catfish, is a diverse order of teleost fishes, represented by about 3790 species, of which 2053 occur in America (Nelson et al. 2016). There is a large concentration of species in the tropical regions of America, Asia, and Africa, with a few families tied to marine water (Burgess 1989). Their most common characteristic is the absence of body scales (although several species have bony plates) and the presence of barbels near the mouth (Santos et al. 2004), but astonishing morphological variation accompanies the species diversity.

Pseudopimelodidae is a monophyletic Neotropical family (Shibatta and Vari 2017; Shibatta et al. 2021; Silva et al. 2021) composed of small to medium‐sized catfish with wide mouths, small eyes covered with skin, robust bodies, small and depressed heads, dorsal fin with a strong spine, and short maxillary and mental barbels (Shibatta 2003). They are widely distributed in South America (Shibatta 2003) and adapted to a benthic lifestyle, with omnivorous to carnivorous feeding habits (Shibatta and van der Sleen 2018).

**Figure 1:**
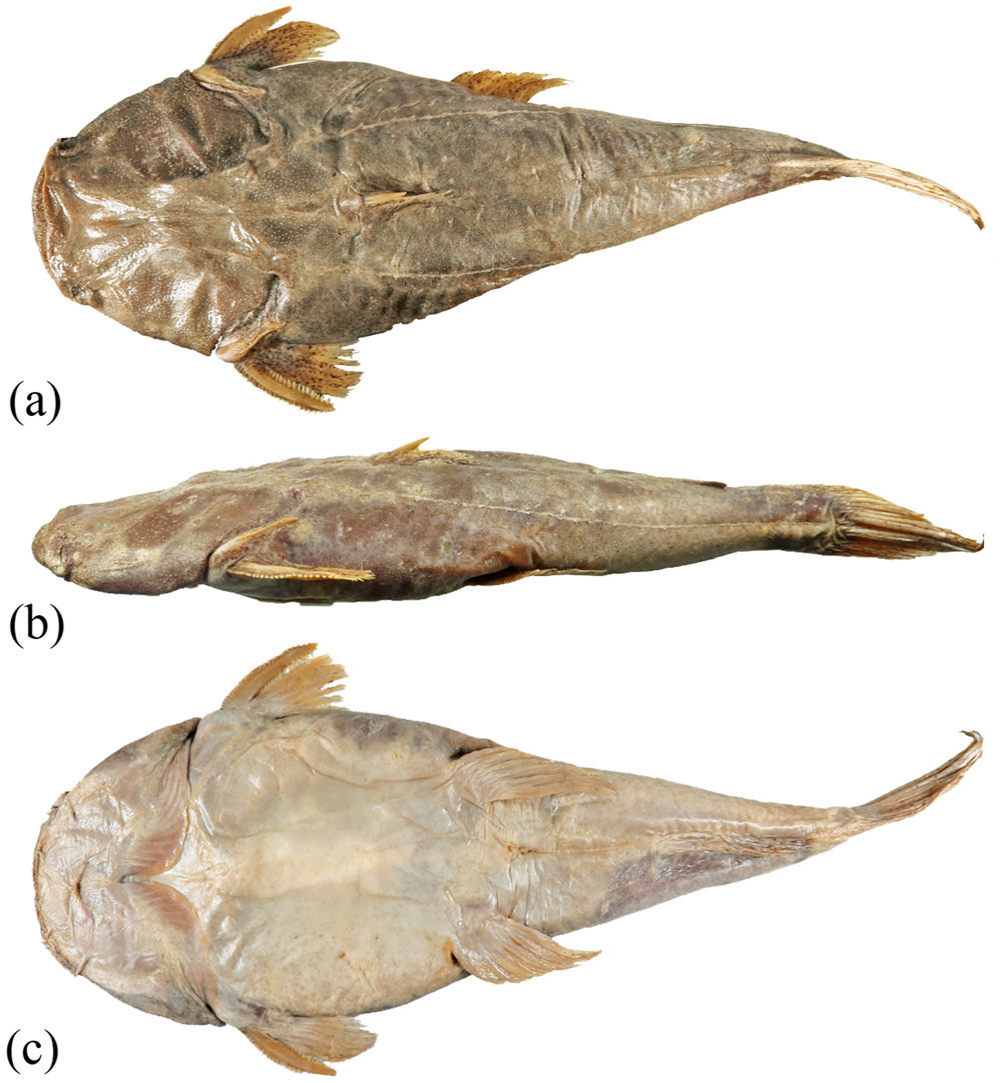
Dorsal (A), lateral (B), and ventral (C) views of *Lophiosilurus alexandri* MZUEL 14134, 227.3 mm SL, São Francisco River basin, Três Marias, Minas Gerais, Brazil.

Among Pseudopimelodidae, the Pacman catfish *Lophiosilurus alexandri* (*L. alexandri*) Steindachner, 1876 (Figure 1) is one of the largest species, approximately 65 cm in total length, with a unique body shape. The head is strongly depressed, the mouth is large, and the body is thicker toward the caudal fin. Its predominant coloration is a greyish‐brown background with some dark‐brown spots. The body shape and the color pattern are advantageous for its psammophilous behavior, which is used when hiding in the sand to ambush the prey (Shibatta 2003). The species is endemic to the São Francisco River basin, commonly known as pacamão, and is endangered with a declining population due to fisheries (Sato et al. 2006).

Based on the external morphology of *L. alexandri*, it is possible to presume that the shapes of muscles might differ slightly from those in other Pseudopimelodidae. Skeletal musculature demonstrates wide diversity among distinct fish lineages, offering a rich source of phylogenetic information (Winterbottom 1974; Datovo and Bockmann 2010; Datovo and Vari 2014; Peixoto and de Pinna 2022). The muscles of the neurocranium play crucial roles in vital fish activities, such as food capture and gill ventilation (Datovo and Vari 2014). Although considerable variation in the dorsolateral head is observed among the main fish lineages, current knowledge about the evolution of this anatomical complex is still limited (Datovo and Vari 2014; Datovo and Rizzato 2018). Studying these structures is essential to understanding how fish have adapted their muscular structures to different ecological niches, whether for efficient swimming, rapid locomotion (Altringham and Ellerby 1999), or predation and feeding (Arce H. 2015; Gidmark et al. 2019).

The myology of Pseudopimelodidae is known only to *Batrochoglanis raninus* (Valenciennes, 1840), as described by Diogo et al. (2004a), evidencing a lack of comparative studies within the family. Thus, this article describes the head musculature of *L. alexandri* and compares it with that of other Pseudopimelodidae. It can also offer an extra number of characters to help understand the functional anatomy and the systematics of the Pseudopimelodidae and the evolution of the Siluriformes.

## 2 Methods and Methods

### 2.1 Material Examined

*Batrochoglanis labrosus* (*B. labrosus*), MZUSP 23864, 1, 75.0 mm HL (head length), Rio Capim, Iguarapé Caranandeua, Pará, Brazil. *L. alexandri*, MZUEL 14135, 1, 70.0 mm HL, São Francisco River basin, Três Marias, Minas Gerais, Brazil. *Lophiosilurus fowleri* (*L. fowleri*), MZUEL 13853, 1, 81.9 mm HL, São Francisco River basin, Três Marias, Minas Gerais, Brazil. *Pseudopimelodus mangurus* (*P. mangurus*), MZUEL 20155, 0103 and, 5741, 3, 28.0–42.3 mm HL, Paranapanema River basin, Paraná, Brazil. *Pimelodus maculatus* (*P. maculatus*), MZUEL 01343, 5, 119.2 mm HL, Ribeirão Três Bocas; Londrina, Paraná, Brazil. *Pimelodus microstoma* (*P. microstoma*), MZUEL 4480, 5, 135.0 mm HL.

Dissections: All soft tissues that could impair the visualization of the musculature (e.g., skin and fascia) were removed using scalpels, tweezers, and scissors under a stereomicroscope. Some muscles were removed to allow the examination of other muscles below. Photographs of the head in dorsal, lateral, and ventral views were taken with a DSLR digital camera, Nikon D5600, 24.2 MP. Specimens of *B. labrosus* Shibatta, 2024, *L. fowleri* Haseman, 1911, *P. mangurus* (Valenciennes, 1835), *P. maculatus* Lacepède, 1803, and *Rhamdia quelen* Quoy and Gaimard, 1824 were included as comparative material. Musculature information on *B. raninus* was obtained from Diogo et al. (2004a), *Heptapterus mustelinu*s from Diogo (2007a), *Lophius piscatorius* (*L. piscatorius*) from Field (1966), and *Chaca bankanensis* from Diogo et al. (2004b). All muscular nomenclatures were updated according to Datovo and Vari (2014) and Datovo and Rizzato (2018).

The method to contrast the muscles from the bones and cartilage was based on Datovo and Bockmann (2010) with a few modifications. The eyes and internal organs of the specimen were removed. The specimen was washed and submerged in tap water for approximately 48 h to hydrate and remove the formaldehyde. The skin was removed, and the cartilage was colored with an acid solution (80 parts 96 GL ethanol + 20 parts glacial acetic acid +10 mg of alcian blue) for approximately 48 h. The specimen was immersed in a saturated borax solution for about 24 h to neutralize the acid solution. Then, the bones were stained in an alcoholic alizarin solution of approximately 10 mg per liter for 3–6 h. Finally, the specimen was preserved in 70% ethanol solution, a concentration that ensured the muscular fibers did not become too fragile or rigid (dehydrated).

Terminology: The terminology used in this study to describe the muscles was based on Datovo and Bockmann (2010), updating the sections of the *adductor mandibulae muscle* according to Datovo and Vari (2014). The cranial osteology nomenclature followed Abrahão and Shibatta (2015) and Shibatta (2019). Although the *adductor arcus palatini* muscle is used in most existing myological studies, it has been applied ambiguously to different muscle components in different fish groups studied. To avoid such inconsistencies in nomenclature, in the present study, the laminar myological component located internally to the floor of the otic cavity is called *adductor hyomandibulae*, following Datovo and Rizzato (2018). The terms “origin” and “insertion” refer to the attachment sites of muscle fibers, with insertion being the site where the muscle attaches to the mobile element or which moves with greater intensity during its contraction. In contrast, the origin is the opposite attachment point, where the muscle connects to the fixed element or moves less during muscular activity.

Phylogenetic analysis: A phylogenetic heuristic analysis was performed with TNT v. 1.6 (Goloboff et al. 2008; Goloboff and Morales 2023) using the “Implicit enumeration” option. Five terminals were analyzed: *P. maculatus* was used as the outgroup to root the tree, and *P. microstoma*, *B. labrosus*, *L. alexandri*, *L. fowleri*, and *P. mangurus* were used as the ingroup. As far as possible, the characters of *Batrochoglanis raninus* were obtained from the literature (Diogo et al. 2004a) and compared with the species dissected in this study. However, the species was not included in the matrix due to the impossibility of obtaining all comparative information. A total of 84 characters were analyzed, 63 of which were based on Shibatta et al. (2021), except four continuous characters, the geometric morphometry characters, and characters 16, 24, 32, 38, 42, 72, and 74, and 21 were obtained from this study. The musculature characters were selected considering variations in size, shape, subdivision, tendons, origin, and insertion. The characters were treated as non‐ordered to avoid any hierarchy between states, considering that the direction of the transformation is unknown. The consistency index, rescaled consistency index, retention index, and homoplasy distribution index were also calculated with TNT v. 1.6 using the script “statsall. run” V.1.3 developed by Peterson L. Lopes, available at Google groups “TNT‐Tree Analysis using New Technology > Per character CI and RI”.

**Ethics statement**

All the specimens studied came from scientific collections and were already dead and preserved in a 70% ethanol solution. Thus, according to Brazilian laws, there is no need for approval of the study by an ethics committee.

## 3 Results

### 3.1 The Dorsolateral Musculature of the *Lophiosilurus alexandri* Head (Figure 2A)

**Figure 2:**
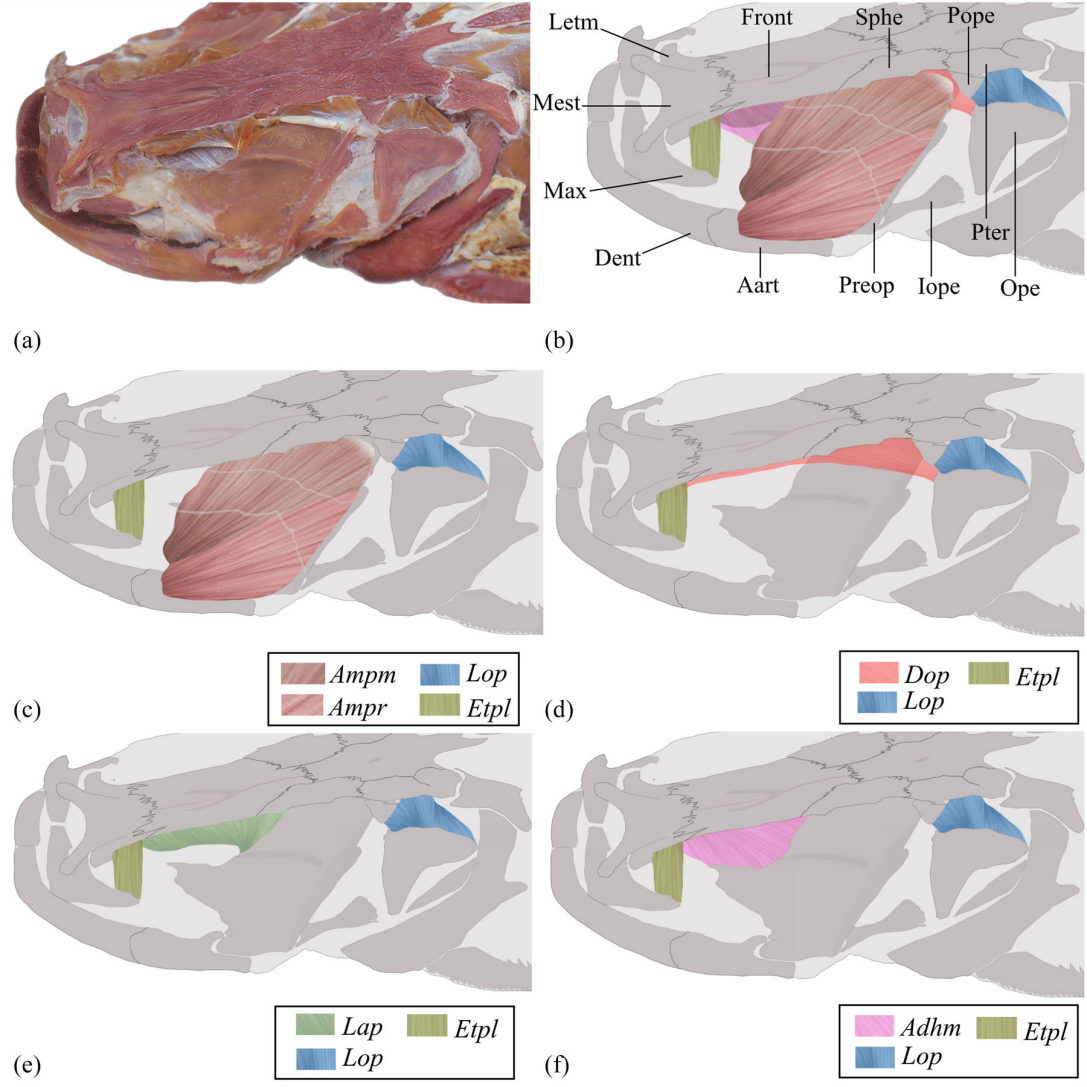
Dorsolateral view of *Lophiosilurus alexandri* head left side, evidencing the musculature (in italics) and bones (regular), MZUEL 14135, 70.0 mm HL. (A) General view of dissected specimen excluding the *adductor mandibulae*. B) Muscles and bones identification. (C–F) Selected muscle evidenced. *Aart, anguloarticular; Adhm, adductor hyomandibulae; Ampm, adductor mandibulae, pars malaris; Ampr, adductor mandibulae, pars rictalis;* Dent, dentary*; Dop, dilatator operculi; Etpl, extensor tentaculi, pars lateralis;* Front, frontal; Iope, interopercle*; Lap, levator arcus palatini; Lope, levator opercle;* Max, maxilla; Mest, mesethmoid; Ope, opercle; Pope, processus opercularis; Preop, preopercle; Pter, pterotic; Sphe, sphenotic.

In the dorsal view of the head, *Lophiosilurus alexandri* has three extensive and conspicuous musculatures (*musculus extensor tentaculi, m. levator arcus palatini*, and *m. adductor mandibulae*). A slightly smaller musculature is the *m. levator operculi* is in the dorsolateral region above the opercle (Figure 2).

#### 3.1.1 Musculus Adductor Mandibulae (Figures 2B–C and 3)

**Figure 3:**
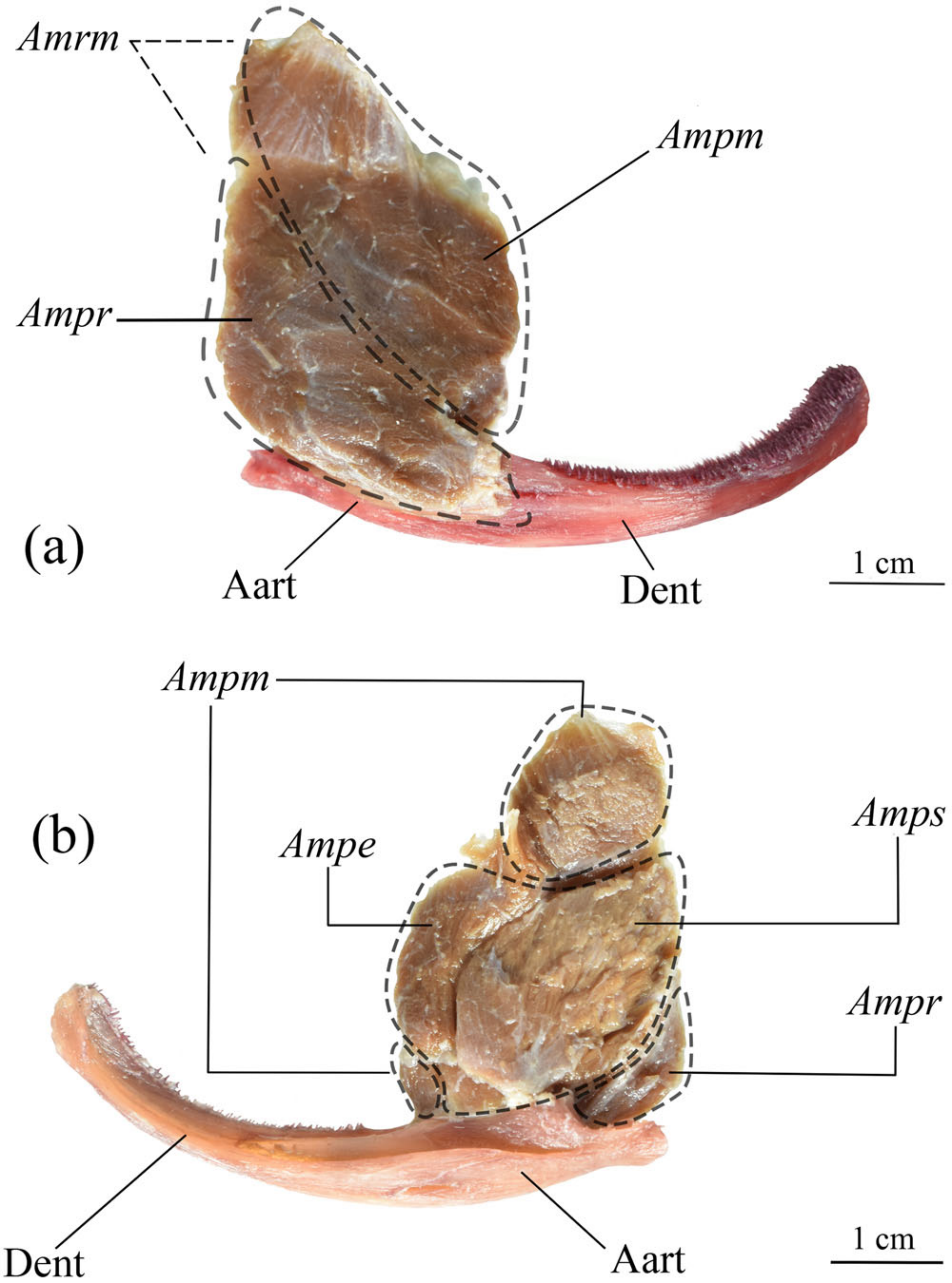
Removed *Lophiosilurus alexandri adductor mandibulae* and ventral mandibulae in (A) lateral and B) medial views evidencing the musculature (italics) and bones (regular); MZUEL 14135, 70 mm HL. Aart, angulo‐articular; Ampe, adductor mandibulae, pars epistegalis; Ampm, adductor mandibulae, pars malaris; Ampr, adductor mandibulae, pars rictalis; Amrm, adductor mandibulae ricto‐malaris (= rictalis + malaris); Amps, adductor mandibulae, pars substegalis; Dent, dentary.

The most voluminous musculature in the head of *L. alexandri* is the *adductor mandibulae*. Its origin is in both the neurocranium and elements of the suspensorium. It is divided into three main segments: *pars malaris, pars rictalis*, and *pars stegalis*. *Pars malaris* and *pars rictalis* have incomplete divisions, forming the *ricto‐malaris* set. *Pars malaris* is the largest muscle of the facial segment, originating in the frontal and sphenotic bones, with its insertion in the dorsal region of the angulo‐articular, with some fibers extending to the posteromedial region of the dentary. The fibers of *pars malaris* are differentiated from *pars rictalis* only near their insertion. The *pars rictalis* is found in the lateral region, ventral to *pars malaris*, originating in the preopercle and quadrate, extending towards the dorsolateral portion of the angulo‐articular, covering it almost completely. Conversely, some muscles may have subdivisions into smaller segments depending on their fiber patterns, such as the *pars stegalis*, which lies medially to the *pars malaris* and is subdivided into *epistegalis* and *substegalis*, originating from the lateral face of the hyomandibula with insertion into the dorsal part of the angulo‐articular. The *adductor mandibulae* has zigzag‐shaped tendinous intersections along the lateral face.

##### 3.1.1.1 Comparative Analysis

The shape of the *adductor mandibulae* in all Pseudopimelodidae is roughly rounded, opposite to the fusiform shape in *P. maculatus* and *P. microstoma*. The absence of tendinous intersections in the *adductor mandibulae pars malaris* complex of *P. maculatus*, *P. microstoma, L. fowleri*, and *P. mangurus* allows us to distinguish them from *L. alexandri* and *B. labrosus*. Also, the *pars malaris* and *pars rictalis* sections in lateral view are more distinct in *P. mangurus* when compared to the other analyzed species, which have a partial division restricted only to their anterior region. Besides, part of the *epistegalis* segment of the *pars stegalis* section, positioned lateral‐dorsally to the *pars malaris*, is visible in lateral view in *P. maculatus*, *B. labrosus*, *L. fowleri*, and *L. alexandri*, but not in *P. mangurus*.

The origin can differ among species, probably adjusting to their head shapes. In *L. alexandri* and *L. fowleri*, the muscle originates from the frontal to the sphenotic, probably because of their more flattened heads. In *P. mangurus*, with a more rounded head, the origin is restricted to the sphenotic and pterotic. Besides, the *adductor mandibulae* in *L. alexandri* do not cover the *dilatator operculi*, unlike all other Pseudopimelodidae. This condition is an autapomorphy of *L. alexandri* but also a reversion since it is shared with Pimelodids.

#### 3.1.2 Musculus Levator Arcus Palatini (Figure 2E)

**Figure 4:**
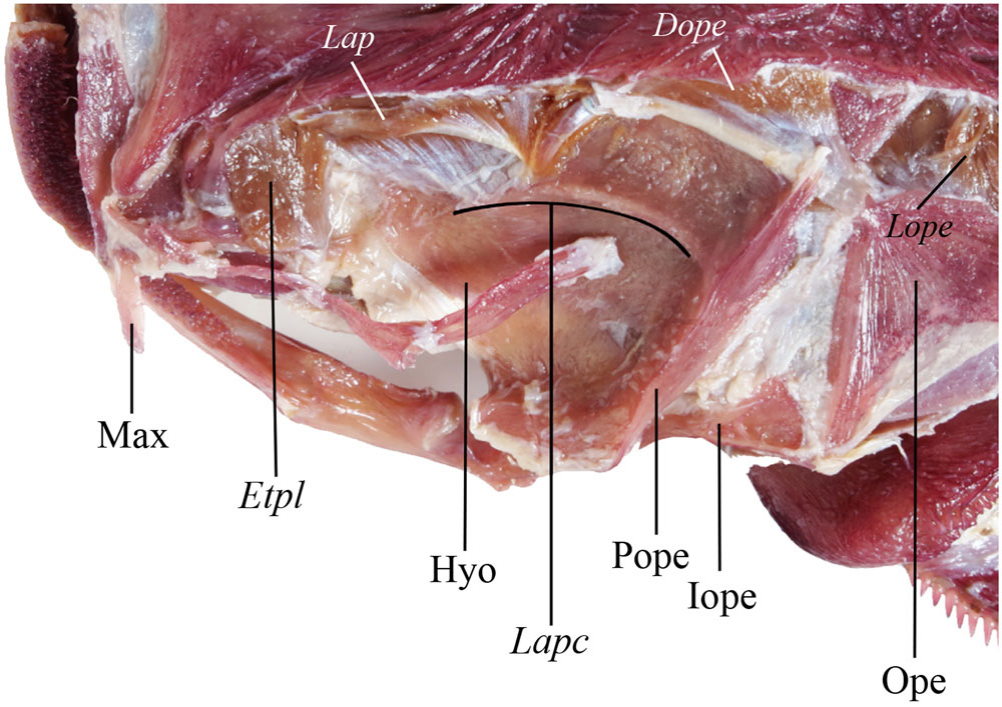
Dorsal view of *Lophiosilurus alexandri* head with *musculus adductor mandibulae* removed to evidence the hyomandibular *levator arcus palatini crest*, MZUEL 14135, 70 mm HL. Bones are in regular fonts, and musculature in italics. *Dope, dilatator oerculi; Etpl, extensor tentaculi, pars lateralis;* Hyo, hyomandibula; *Lap*, *levator arcus palatini*; *Lapc, levator arcus palatini* crest; Max, maxilla; Ope, opercle; Pope, preopercle.

In *L. alexandri*, the muscle has an obtuse triangle shape, occupying a wide area at its origin and converging at a common point. It is located medially to the *adductor mandibulae* complex and comprises two parts (anterior and posterior) with distinct fiber patterns. The origin of the anterior part is located along the entire lateral margin of the frontal bone, and it has fibers arranged diagonally in the lateral view. The insertion of the anterior *levator arcus palatini* is mainly concentrated at a single point of the anterior region of the hyomandibular crest (or *levator arcus palatini* crest; Figure 4). The posterior *levator arcus palatini* differs visually from the anterior one because it is smaller and has a fan shape. Its origin is both in the posterior portion of the frontal bone and the lateral margin of the sphenotic, with the fibers arranged both vertically and diagonally, converging to the same insertion point of the anterior *levator arcus palatini* on the anterior region of the *levator arcus palatini* crest.

##### 3.1.2.1 Comparative Analysis

In *L. fowleri*, the anterior *levator arcus palatini* resembles *L. alexandri* having a triangular shape, with its anterior region originating in the final area of the lateral ethmoid and the entire lateral margin of the frontal, not including the sphenotic, and the insertion at a single point in the anterior region of the *levator arcus palatini* crest. In *P. maculatus*, the origin of the *levator arcus palatini* does not include the lateral ethmoid, beginning in the frontal bone, but contains the sphenotic posteriorly. The posterior part of the *levator arcus palatini* insertion of *L. fowleri* differs from *L. alexandri* since it occurs from the middle of the hyomandibula dorsal margin to the anterior region of the *levator arcus palatini* crest. *P. mangurus* differs in the shape of the *levator arcus palatini* muscle, in which the division of anterior and posterior parts is not conspicuous, giving the shape of an irregular trapezoid to the set. Its origin occurs at the beginning of the lateral ethmoid, margins of the frontal, and sphenotic, with short fibers of the posterior region gradually extending anteriorly. The insertion extends along the entire medial margin to the high laminar *levator arcus palatini* crest, which covers the muscle's insertion. This condition differs from that of *P. maculatus*, *P. microstoma, B. labrosus*, and *Lophiosilurus* species, in which the crest is shallow and does not cover the muscle insertion (Figure 4). In *B. labrosus*, the origin includes the lateral ethmoid anteriorly and the sphenotic posteriorly. The length of the *levator arcus palatini* from origin to insertion is another informative character: minor in *P. maculatus*, median in *P. mangurus*, and long in *Lophiosilurus* species and *B. labrosus*.

#### 3.1.3 Musculus Adductor Hyomandibulae (Figure 2F)

The *adductor hyomandibulae* of *L. alexandri* is a laminar muscle positioned medially to the *levator arcus palatini* and the *adductor mandibulae* (Figures 2 and 4). It is a muscle that lines the floor of the eye socket. It originates internally in the orbitosphenoid and pterosphenoid, extending to the dorsal margin of the hyomandibula and inserting in the dorsal portion of the metapterygoid and entopterygoid.

##### 3.1.3.1 Comparative Analysis

The analyzed species have the same *adductor arcus palatini* origin and insertion pattern. However, in *P. maculatus*, *P. microstoma*, *L. alexandri*, and *L. fowleri*, the *adductor arcus palatini* occupies a large area because it has a broad space for the development of the fiber due to the bone morphology where this muscle is attached. In contrast, *P. mangurus* and *B. labrosus* have a reduced space for developing this muscle, which restricts its growth in these species. It is observed that a more extensive and flatter hyomandibular bone, as in *L. alexandri* and *L. fowleri*, allows more space for an enlarged muscle, where part of its anterior portion is visible without muscle removal, even though it is medial to the *levator arcus palatini*. It is the same in *P. maculatus* and *P. microstoma*, but in *P. mangurus* and *B. labrosus*, the *adductor arcus palatini* is hidden by the *levator arcus palatini*.

#### 3.1.4 Musculus Levator Operculi (Figure 2)

In *L. alexandri, levator operculi* is structured in two triangular parts; one is a mirror image of the other. The anterior portion originates in the *processus opercularis* and pterotic. Its insertion occurs at a single point of the anterodorsal region of the opercle. On the other hand, the origin of the posterior portion is restricted to the posterolateral margin of the pterotic, and its fibers diverge vertically for insertion in the posterodorsal portion of the opercle.

##### 3.1.4.1 Comparative Analysis

The origin of the *levator operculi* of *L. fowleri*, *P. mangurus*, and *P. microstoma* exhibits the same characteristics observed in *L. alexandri*, originating in the *processus opercularis*. This region is exposed and can be visualized laterodorsally. In *P. maculatus* and *B. labrosus*, the *levator operculi* originates in the pterotic, with its fibers covering the *processus opercularis*. The insertion of the anterior portion of the *levator operculi* occurs in the posterodorsal region of the hyomandibula and is visible laterodorsally in *L. alexandri* and *L. fowleri*. However, *L. fowleri* differs in that the fibers of the anterior *levator operculi* originate at the posterodorsal of the preopercle.

#### 3.1.5 Musculus Dilatator Operculi (Figure 2D)

*L. alexandri* has a long *dilatator operculi* muscle with the origin in the posterior portion of the lateral ethmoid and all the extension from the frontal to the sphenotic. The insertion occurs through the transverse fibers when viewed laterally, converging towards the anterodorsal condyle of the opercle. It is partially covered by the *levator arcus palatini* and *adductor mandibulae*, but its final portion is uncovered and can be seen in lateral view.

##### 3.1.5.1 Comparative Analysis

*L. alexandri and L. fowleri* have the origin of the *dilatator operculi* muscle beginning in the lateral ethmoid bone, while in *P. maculatus*, *P. microstoma, P. mangurus*, and *B. labrosus*, it is in the frontal bone. The posterior portion of the *dilatator operculi* origin is in the sphenotic bone in *L. alexandri*, *P. mangurus*, *B. labrosus*, and *P. maculatus*, while in *L. fowleri*, it is in the pterotic bone. The insertion of this muscle is similar among the analyzed species, being directed to the anterodorsal condyle of the opercle. However, this muscle is visible laterally only in *L. alexandri* and *P. maculatus*, while the *adductor mandibulae* in the other species hides it.

#### 3.1.6 Musculus Adductor Operculi

A triangle‐shaped muscle in *L. alexandri* lies medially to the *levator operculi*. Its visualization is not possible in lateral view, originating in the medial portion of the posttemporal and pterotic. Its fibers are arranged vertically and inserted into the dorsomedial margin of the opercle.

##### 3.1.6.1 Comparative Analysis

The fibers of the *adductor operculi* follow the variation of the opercle shape, being longer or shorter. In an opercle with a larger dorsal region, the fibers are arranged more diagonally to insert into its posterior end. In species with a smaller opercle, the fibers do not need to be as long and may have vertical or slightly transverse patterns. Although the *adductor operculi* may vary among species, the positions of origin and insertion of the fibers of the analyzed species are the same, with no significant differences. In *L. alexandri*, the cranial morphology and arrangement of bones, such as the opercle, favor the presence of more elongated *adductor operculi* muscle fibers. In contrast, in *L. fowleri*, the *adductor operculi* fibers are of intermediate length. Already in *P. maculatus*, *P. microstoma*, and *P. mangurus*, the *adductor operculi* fibers are smaller due to the short distance between the opercle and the bones of the neurocranium.

#### 3.1.7 Musculus Extensor Tentaculi (Figures 2 and 5)

**Figure 5:**
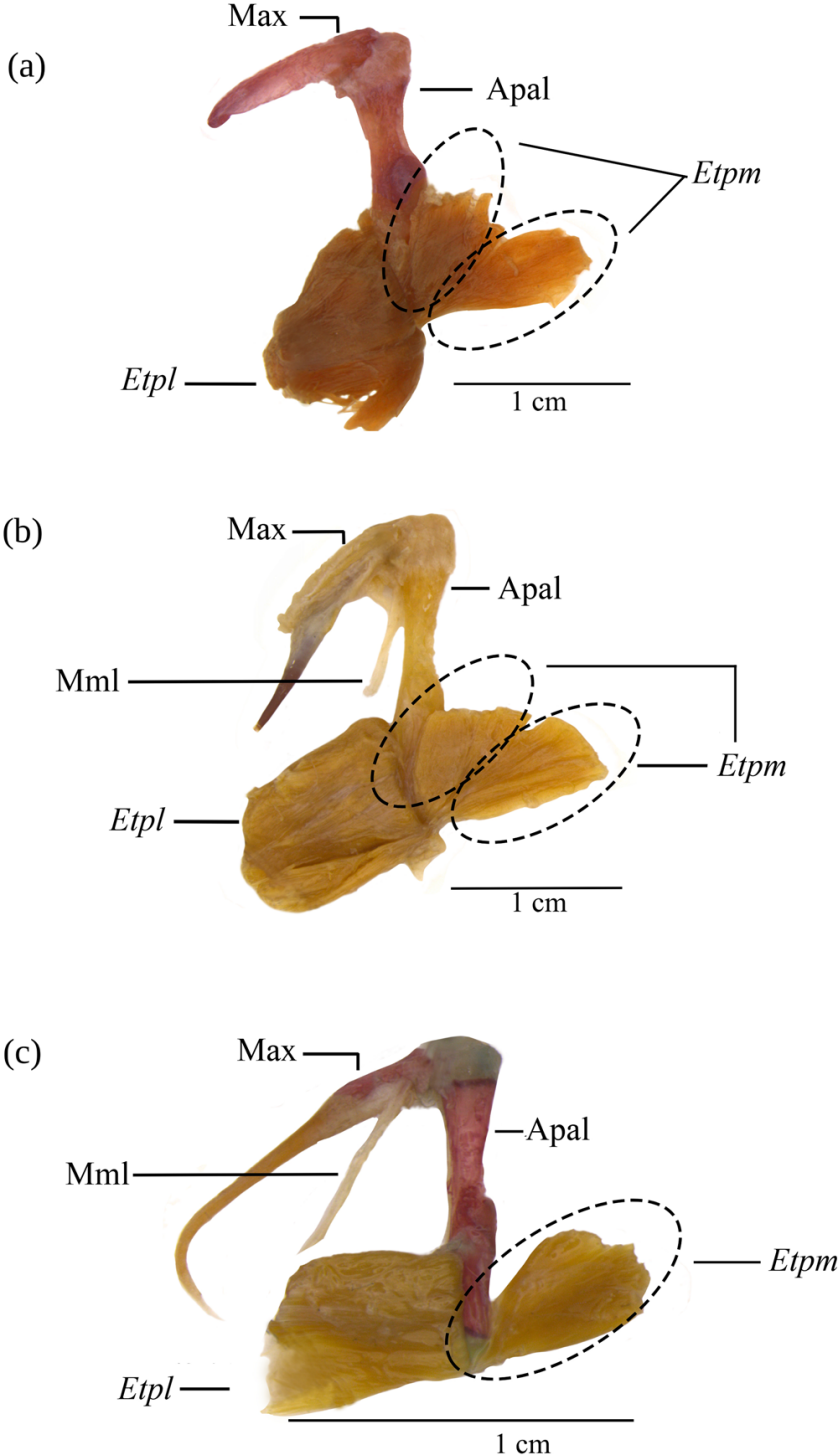
*Extensor tentaculi, pars lateralis* (*Etpl*), and *pars medialis* (*Etpm*) of *Lophiosilurus alexandri* (A), *Lophiosilurus fowler*i (B), and *Pseudopimelodus mangurus* (C) encircling the fiber patterns with dashed lines*. Etpl* turned laterally to expose the *Etpm*. Bones are in regular fonts, and musculature in italics. Apal, autopalatine; Max, maxilla; Mml, maxillo‐mandibular ligament.

In *L. alexandri*, this muscle is divided into two sections, with the first being the *extensor tentaculi, pars lateralis*, which consists of a single myological unit with fibers arranged vertically in a lateral view. Its fibers originate in the posterolateral region of the lateral ethmoid and are inserted in the posterodorsal portion of the autopalatine. The *extensor tentaculi, pars medialis* fibers present two distinct patterns of orientation (Figure 5). The anterior region has a roughly isosceles triangle shape with transverse fibers originating in the anteroventral portion of the lateral ethmoid. It is inserted at a point in the posteromedial region of the autopalatine. The posterior region also has a triangle shape, with fibers arranged vertically, originating from the orbitosphenoid and the posterolateral portion of the lateral ethmoid, and insertion converging to a point in the posteromedial portion of the autopalatine.

##### 3.1.7.1 Comparative Analysis

The *extensor tentaculi, pars lateralis* and *medialis* of both *Lophiosilurus* species analyzed exhibit the exact characteristics of origin and insertion in their muscular set and the fiber pattern observed in the *pars medialis*, which allows them to be distinguished into two parts. *P. maculatus*, *P. microstoma*, and *B. labrosus* also have two distinguishable parts in this muscle. However, *P. mangurus* differs by presenting the *extensor tentaculi pars medialis*, composed of a fiber pattern that remains a single myologic unit.

#### 3.1.8 Musculus Protractor Hyoidei (Figure 6)

**Figure 6:**
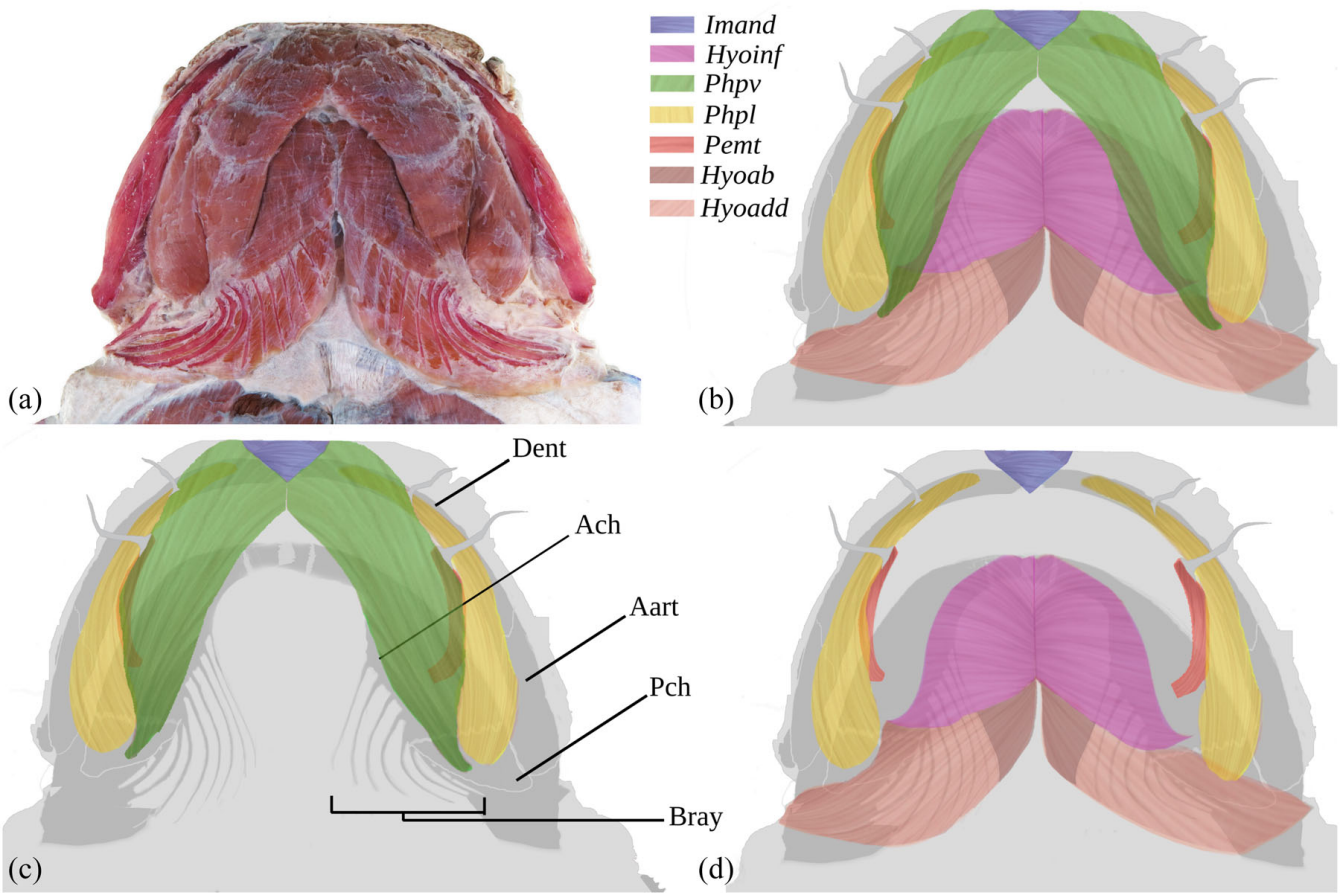
*Lophiosilurus alexandri* head ventral musculature (italics) and bones (regular); MZUEL 14135, 70 mm HL. (A) General view of dissected specimen. (B) All colored musculature. (C) Selected musculature and bones. (D) Selected musculature. Aart, angulo‐articular; Bray, branchiostegal rays; Dent, dentary; *Hyoab*, *Hyohyoideus abductor*; *Hyoadd*, *Hyohyoideus adductor*; *Hyoinf*, *Hyohyoideus inferior*; *Imand*, *Intermandibularis*; *Pemt*, *Protractor externi mandibularis tentaculi*; *Phpl*, *Protractor hyoideus, pars lateralis*; *Phpv*, *Protractor hyoideus, pars ventralis*.

In *L. alexandri*, two portions of the *protractor hyoidei* were identified: the *Protractor hyoideus, pars lateralis*, and the *Protractor hyoideus, pars ventralis*. The *pars lateralis* exhibits a drop‐like morphology, with its narrower anterior part gradually expanding to a more robust shape in the posterior region. Its origin occurs in the anteromedial portion of the dentary, extending until its insertion in the posterior ceratohyal and located laterally to the *pars ventralis*. On the other hand, the pars ventralis originates in the dentary and is inserted into the posterior portion of the posterior ceratohyal.

##### 3.1.8.1 Comparative Analysis

In *P. mangurus*, *B. labrosus, P. maculatus*, and *P. microstoma*, the *pars lateralis* originates in the median region of the dentary, while in the analyzed species of *Lophiosilurus*, the origin is in the anterior region of the dentary.

#### 3.1.9 Musculus Hyohyoideus Inferior (Figure 6B,D)

In *L. alexandri*, it is a robust muscle located in the ventral region of the neurocranium and partially hidden at its lateral edge by the *protractor hyoideus pars ventralis*. Its origin occurs anteriorly in the medial portion of the parurohyal, in the anterior ceratohyal, and at the base of the first six branchiostegal rays.

##### 3.1.9.1 Comparative Analysis

Although the characteristic attachment of this muscle is similar among the species examined, there are notable differences. In *L. alxandri* and *L. fowleri*, the muscle has a larger area visible ventrally; in *P. mangurus*, this area is smaller. Furthermore, in *P. mangurus*, the muscle is attached to the base of the first five rays, unlike *Lophiosilurus* species, *B. labrosus, P. maculatus*, and *P. microstoma*, where it is connected to the base of the first six branchiostegal rays.

#### 3.1.10 Musculus Hyohyoideus Abductor

A muscle that extends from the first branchiostegal ray to a fibrous set (aponeurosis) medially to the *hyohyoideu*s *inferior*, aggregated to tendinous fibers connected to the posterior portion of the paruroyal. The characteristics of this muscle in all the species analyzed do not differ, with the connection between the muscle and tendons and the adhesion to the parurohyal bone remaining the same.

#### 3.1.11 Hyohyoideus Adductor (Figure 6B,D)

It is the muscle located in the ventral region of the head that connects the branchiostegal rays. Its fibers are arranged in a diagonal direction and connect the first five branchiostegal rays in *P. maculatus*, *P. microstoma*, and *P. mangurus*. In *L. alexandri* and *L. fowleri*, this muscle connects the first eight branchiostegal rays, decreasing in size through the remaining rays until they become inconspicuous because of the close distance of the rays.

#### 3.1.12 Musculus Intermandibularis (Figure 6)

In all species examined, a small muscle in the antero‐ventral region joins the mandibles. The semicircular muscle connects its dorsal surface to the dentary's anteromedial region. Its ventral surface is intertwined with fibers belonging to the *protractor hyohyoideus*, *pars ventralis*.

Attachment points or fiber patterns do not differ between the species analyzed. However, the fusiform shape of the *intermandibularis* in *P. mangurus*, *P. maculatus*, and *P. microstoma* differs from the semicircular shape of the *Lophiosilurus* species and *B. labrosus*.

#### 3.1.13 Musculus Protractor Externi Mandibularis Tentaculi (Figure 6D)

**Figure 7:**
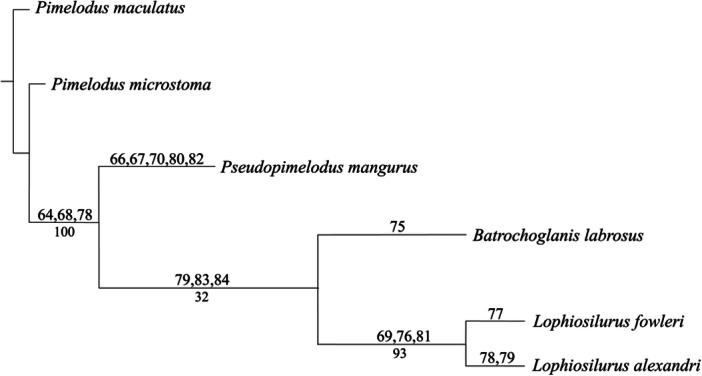
Phylogenetic tree from Pseudopimelodidae musculature analysis. Tree score = 30 steps. The numbers above the branches correspond to the synapomorphies, and the numbers under the branches are the percentage values of Bootstrap.

In all analyzed species, it is a muscle between the ventral and lateral parts of the *hyohyoidei protractor* (Figure 7). In B*. labrosus*, *L. fowleri, and L. alexandri*, the muscle originates in the central portion of the anterior ceratohyal and inserts medially directly into the mental barbels involved in the movements of the barbels. In *P. maculatus, P. microstoma*, and *P. mangurus*, this muscle originates in the final portion of the anterior ceratohyal, which makes its fibers longer.

This muscle exhibits a reduced dimension in *L. alexandri, L. fowleri*, and *B. labrosus*, compared to a moderate size in *P. mangurus*, *P. maculatus*, and *P. microstoma*. Although its location remains constant among species, its origin in *P. mangurus* is in the posterior margin of the anterior ceratohyal, extending to its insertion in the anterodorsal region of the cartilage associated with the mental barbels; a cartilage is not present in the *Lophiosilurus* species analyzed.

##### 3.1.13.1 Phylogenetic Analysis

Twenty‐one myological characters were used for phylogenetic analysis. Supplementary online material includes more information on the character codification (Supporting Information Table S1), the data matrix (Supporting Information Table S2), the list of synapomorphies, and the phylogenetic indexes (Supporting Information Tables S3–S8). One of the most parsimonious trees was obtained (Figure 7) with 108 steps, a consistency index of 0.82, a rescaled consistency index of 0.62, a retention index of 0.76, and a homoplasy distribution index of 0.07. The monophyly of the clade formed by *L. alexandri* and *L. fowleri* was recovered with three synapomorphies (characters [chars.] 69, 76, and 80) and a bootstrap frequency of 93%. *L. alexandri* presented two autapomorphies (chars. 78 and 79) and *L. fowleri* one (char. 77). *B. labrosus*, with one autapomorphy (char. 75), was the sister of the *Lophiosilurus* species. This clade presented three synapomorphies (chars. 79, 83, and 84) and a bootstrap frequency of 33%. *P. mangurus*, with five autapomorphies (chars. 66, 67, 70, 80, and 82), was the sister group of *B. labrosus, L. fowleri*, and *L. alexandri*, forming the Pseudopimelodidae clade. The support of this clade reached 78% in bootstrap frequency.

The clade *B. labrosus*, *L. fowleri*, and *L. alexandri* synapomorphies were char. 79 (CI = 1.0; RI = 1.0), *adductor operculi*, large to medium (vs. small); char. 83 (CI = 1.0; RI = 1.0), *intermandibularis*, semicircle shape (vs. fusiform); char. 84 (CI = 1.0; RI = 1.0), *protractor externi mandibularis tentaculi*, reduced (vs. moderate).

The synapomorphies of the *L. alexandri* and *L. fowleri* clade were char. 69 (CI = 1.0; RI = 1.0), *levator arcus palatini*, posterior of origin not including the sphenotic (vs. including); char. 76 (CI = 1.0; RI = 1.0), *dilatator operculi*, beginning of origin in the lateral ethmoid (vs. in the frontal bone); char. 81 (CI = 1.0; RI = 1.0), *protractor hyoideus*, *pars lateralis*, origin on the anterior region of the dentary (vs. median portion of dentary).

*Lophiosilurus alexandri* autapomorphies were char. 78 (0.5; RI = 0.5), *dilatator operculi*, exposition in lateral view, covered by the *adductor mandibulae* (vs. not covered; reversal in *L. alexandri*); char. 79 (CI = 1.0; RI = 1.0), *adductor operculi*, large (vs. small or medium).

*Lophiosilurus fowleri* presented one autapomorphy: char. 77 (CI = 1.0; RI = 1.0), end of *dilatator operculi* origin on the pterotic bone (vs. on the sphenotic bone).

The autapomorphies of *P. mangurus* were char. 66 (CI = 1.0; RI = 1.0), *adductor mandibulae*, *pars malaris* and *pars rictalis*, lateral view, partial division not restricted to their anterior region (vs. restricted); char. 67 (CI = 1.0; RI = 1.0), *adductor mandibulae*, *pars stegalis*, *epistegalis*, not evident (vs. evident); char. 70 (CI = 1.0; RI = 1.0), *levator arcus palatini*, insertion, covered by a crest (vs. not covered); char. 80 (CI = 1.0; RI = 1.0), *extensor tentaculi*, *pars medialis*, one part (vs. two); char. 82 (CI = 1.0; RI = 1.0), *hyohyoideus inferior*, covering the first five branchiostegal rays (vs. six branchiostegal rays).

TNT recovered three characters as synapomorphies of the Pseudopimelodidae clade: char. 64 (CI = 1.0; RI = 1.0), *adductor mandibulae*, rounded shape (vs. elongated); char. 68 (CI = 1.0; RI = 1.0), *levator arcus palatini*, origin, lateral ethmoid included (vs. not included); char. 78 (0.5; RI = 0.5), *dilatator operculi*, not exposed in lateral view, covered by the *adductor mandibulae* (vs. not covered; homoplastic with *P. maculatus*).

The character 71 (CI = 1.0; RI = 1.0), *levator arcus palatini*, length from origin to insertion, was not considered as synapomorphy and is multistate, with one of state exclusive to *P. mangurus* and the other to the clade *B. labrosus*, *L. alexandri*, and *L. fowleri*; char. 74 (CI = 0.33; RI = 0.33), *levator operculi*, origin including the pterotic and the *processus opercularis* was homoplastic among *P. microstoma*, *P. mangurus*, and *L. alexandri*, or including only the pterotic was homoplastic among *P. maculatus* and *B. labrosus*, or the pterotic and posterodorsal end of the preopercle are exclusive of *L. fowleri*. Three other characters were homoplastic and were not considered synapomorphies: char. 65(CI = 0.5; RI = 0.0), *adductor mandibulae*, *pars malaris* tendinous intersection, char. 72 (CI = 0.5; RI = 0.5), *adductor arcus palatini* covered by the *levator arcus palatini*, and char. 73 (CI = 0.5; RI = 0.5), *adductor arcus palatini* size.

## 4 Discussion

The comparative analyzes revealed characteristics of *Lophiosilurus alexandri* that differ from the other species analyzed in several head muscle groups with respect to the origin and insertion or the shape and arrangement of the fibers. The tree obtained with head musculature shows phylogenetic relationship similarities with a tree obtained by a more encompassing morphological analysis (Shibatta et al. 2021). For instance, *L. alexandri* and *L. fowleri* are a sister group, *B. labrosus* is the sister of this group, and *P. mangurus* is the sister of all. The consistency and retention index values of 0.82 and 0.76, respectively, evidence some homoplasies, but also that the musculature characters are informative. Therefore, the phylogenetic analysis allowed us to understand the evolution of several sets of musculature, as presented below.

### 4.1 The Evolution of Adductor Mandibulae

In Pseudopimelodidae, the *adductor mandibulae* occupy a considerable head area and evolved to a rounded shape instead of an elongated shape, as in Pimelodidae.

A character that evolved only in *L. alexandri* is the presence of tendinous intersections along the *adductor mandibulae* complex's lateral face. Although the precise function of this characteristic remains undefined, previous studies on striated musculature suggest that tendinous intersections are present in muscles interconnected to a tendon or aponeurosis, causing the segmentation of a muscle into smaller parts (Datovo and Vari 2013). Besides, changes in subdivision and position in parts of the *adductor mandibulae* evolved differently in *P. mangurus*. For instance, the partial subdivision of the *pars malaris* and *pars rictalis* extends to the median area of the *adductor mandibulae* complex (vs. restricted to the anterior region, identified as *ricto‐malaris*, in *L. alexandri*), and the *epistegalis* is not evident (vs. evident in *L. alexandri*).

The high development of the *adductor mandibulae* in Pseudopimelodidae effectively helps to capture the prey. The success of this process is related to the synergistic action of the *adductor mandibulae* with the ligaments between the lower jaw and the suspensory, the robust *intermandibularis*, the *protractor hyoideus*, and the *levator operculi* generating a negative pressure in the oral cavity when the mouth opens (Adriaens et al. 2001). In many vertebrates, the force generated by the muscles responsible for the animal's bite is related to the geometry of skull structures (Anderson et al. 2008). This dependence influences the development of more commonly used muscles and other head parts, determining the head shape. However, the opposite can be observed in different groups of fish. For example, in some Trichomycteridae that are parasites or feed on worms or debris, the musculature is not developed (e.g., Datovo and Bockmann 2010), making room to establish other organs like a more voluminous eye.

### 4.2 The Evolution of Levator Arcus Palatini

Including the lateral ethmoid in the origin of the *levator arcus palatini* evolved in the Pseudopimelodidae, while in *Pimelodus*, the origin begins medially in the frontal bone. In fishes that have a large oral cavity, such as Pseudopimelodidae, an elongated morphology of the *levator arcus palatini* is expected, as documented in Auchenipteridae by Sarmento‐Soares and Porto (2006). In *Lophiosilurus alexandri*, the origin of this muscle extends to the anterior region of the neurocranium, corroborating that hypothesis.

A deep *levator arcus palatini* crest of the hyomandibula evolved in *P. mangurus*, covering the insertion of the muscle *levator arcus palatini*. The *levator arcus palatini* plays a crucial role in the lateral expansion of the oral cavity through suspensorium abduction (Anderson and Westneat 2007; Brocklehurst et al. 2019), being intrinsically linked to kinematic control of the mouth (speed of muscle execution), as observed by Day et al. (2015). Such control may be especially relevant during asymmetric attacks when fish attempt to capture more elusive prey, as Liem (1980) discussed. However, the role of this insertion protection in *P. mangurus* still needs further investigation.

### 4.3 The Evolution of the Opercle Musculature

The evolution of the two Pseudopimelodidae subfamilies is reflected in the *adductor operculi* muscle. In Batrochoglaninae, this muscle seems to be more extensive than in Pseudopimelodinae. It is noteworthy that Batrochoglaninae has a more depressed and broader head than Pseudopimelodinae, and the opercle tends to be farther away from the axis of the head, causing elongation of the *adductor operculi* fibers. In *L. alexandri*, the *adductor operculi* is even larger, which can be related to the depressed head and evidence of the greater strength in the opercle closing compared to the other species.

Maybe the *dilatator operculi* covered by the *adductor mandibulae*, making it invisible in lateral view, is also a Pseudopimelodidae synapomorphy. However, it is homoplastic with *P. maculatus* but not with *P. microstoma*, deserving of further analysis, including more Pimelodidae species. Additionally, there are other distinct morphological variations in the Pseudopimelodidae members compared to different groups of fish. For example, the *levator operculi* in most catfish originates exclusively from the pterotic (Geerinckx and Adriaens 2008; Sarmento‐Soares and Porto 2006; Arce H. 2015). Even so, in *L. alexandri*, *P. mangurus*, and *Batrochoglanis raninus* (Diogo et al. 2004a), some fibers originate from the *processus opercularis*, indicating a putative synapomorphy of the group.

### 4.4 The Evolution of Extensor Tentaculi

The *extensor tentaculi*, *pars medialis*, presents only one part, not two, in *P. mangurus*. Differences in the fiber pattern organization of this muscle were observed between species of *Lophiosilurus* and *P. mangurus*. The *extensor tentaculi* corresponds to the muscular set responsible for the movement of the maxillary barbels. Its function is related to the abduction of the barbels, and the maxillo‐mandibular ligament appears as an antagonist, acting as an adductor (Royero et al. 1997). Geerinckx et al. (2009) proposed a nomenclature for the bands, with *pars lateralis* being more distal and *pars medialis* being more proximal to the neurocranium axis. The literature suggests that one or two myologic components of this muscle are plesiomorphic. However, upon analyzing the *pars medialis* of *L. alexandri* and *L. fowleri*, distinct patterns in fiber organization were observed, allowing us to distinguish them into two subdivisions. Although some fibers exhibit a nonorganized pattern suggesting these subdivisions, they cannot be categorized as distinct bands, as seen with *pars medialis rostralis* and pars *medialis caudalis* in Pimelodidae, Bagridae, or Loricariidae (Adriaens and Verraes 1997; Diogo and Chardon 2000), where the morphology, patterns, and often attachment sites differ. In contrast, *P. mangurus* displays a plesiomorphic condition when the pattern of fiber organization of the *pars medialis* results in a single myology unit. Regarding the *extensor tentaculi, pars lateralis*, no significant morphological differences or fiber patterns were identified among the studied species.

### 4.5 The Evolution of Retractor Tentaculi

Furthermore, the absence of the *retractor tentaculi* in the Pseudopimelodidae and Pimelodidae studied is noteworthy. This muscle is responsible for the adduction of the maxillary barbels. It comprises distinct and exclusive elements of the order Siluriformes and is always referred to as any myologic component originating in the suspensorium and inserted in the jaw (Winterbottom 1974; Diogo et al. 2003). It may be the result of the differentiation of the *adductor mandibulae*, *pars stegalis* in some Siluriformes (Diogo and Chardon 2000; Diogo et al. 2003), *pars rictalis* or *pars malaris* in other groups (Diogo and Chardon 2000). The maxillary barbel adduction is mediated mainly by the stretching of the maxillo‐mandibular ligament and can be divided into two cycles. In the first cycle, this ligament features regions of crimped (wavy) fibers within its structure. During the barbel abduction, this ligament is tensioned due to its elastic properties, enabling it to store energy and release it when the *extensor tentaculi* muscle ceases its action. The second part of the cycle involves restoring the barbel to its original position, facilitated by releasing stored energy in the maxillo‐mandibular ligament (Royero et al. 1997). However, due to its absence in the Pseudopimelodidae and Pimelodidae examined, the function of tentacular adduction is due, at least partially, to different structures, such as the elevation of the mandible (Royero et al. 1997; Diogo et al. 2003) and the maxillo‐mandibular ligament, as was observed in *Parauchenipterus galeatus* Linnaeus 1766 by Royero et al. (1997). These characteristics are present in all individuals analyzed, with no significant differences.

The absence of the *retractor tentaculi* could be a putative synapomorphy of Pseudopimelodidae and Pimelodidae within Pimelodoidea since this muscle is present in *Heptapterus mustelinus* (Valenciennes, 1835) (Diogo 2007a). However, Buitrago (2006) observed the presence of the *retractor tentaculi* in the Pimelodidae *Pseudoplatystoma fasciatus* and *Hemisorubim platyrhynchos*, showing that the character states are variable in Pimelodidae and the absence within the family may have evolved independently. However, the absence of the character state in all the Pseudopimelodidae analyzed shows that the trait can be considered a synapomorphy of the group since the presence of the *retractor tentaculi* is the plesiomorphic state.

### 4.6 The Evolution of Ventral Head Musculature

In the ventral region of the head of a catfish, the *protractor hyoideus* may have three distinct components (Diogo et al. 2004a). However, in *L. alexandri*, the *pars dorsalis* segment of the *protractor hyoideus* muscle was not identified. According to Diogo's studies on the myology of the cephalic region of *B. raninus* (Diogo et al. 2004a) and *Heptapterus mustelinus* (Diogo 2007a), this muscle originates in the ceratohyal and inserts on the anterodorsal surface of the dentary. Therefore, this muscle may also be present in *L. alexandri*, but it is not easy to find due to its internal location.

The *hyohyoideus inferior* may be a representative of the two subfamilies' evolution since in *P. mangurus*, a Pseudopimelodinae, it covers only five branchiostegal rays, unlike in the Batrochoglaninae species analyzed, which are characterized by having the first six branchiostegal rays covered. However, more Pseudopimelodinae must be analyzed. Besides, in Batrochoglaninae, the *intermandibularis* has a semicircle shape and is not fusiform, and the protractor *externi mandibularis tentaculi* is reduced if compared to *P. mangurus*.

### 4.7 Analysis of Muscular Convergences in Different Species With Similar External Gross Morphologies

Analyzing each species' morphological and anatomical characteristics and a detailed investigation of its natural history allows us to identify associative patterns and infer some of its habits and lifestyle. Different species may present convergences in external gross morphology attributed to their lifestyle, foraging strategies, or environmental pressures (Douglas and Matthews 1992). However, when considering the cranial morphology and behavior of *L. piscatorius* Linnaeus, 1758, a bottom‐dweller predator with an ambush strategy and camouflages in the substrate (Field 1966; Negzaoui‐Garali et al. 2008), and comparing it with *L. alexandri*, significant differences in muscle arrangements are observed.

In *L. piscatorius*, the *adductor mandibulae* is approximately rectangular, running longitudinally in the ventral region of the head (Field 1966), leaving ample space in the dorsal region for the development of the *adductor hyomandibulae*. The strong *adductor hyomandibulae* is crucial to elevating the oral cavity for prey‐sucking (Lauder 1983). Therefore, *Lophiosilurus alexandri* may have a more powerful bite than *Lophius* and use the strong hyoid musculature for suction.

Another muscle that stands out in differentiating between both species and is directly related to respiratory processes and feeding is the *levator arcus palatini*. In *L. alexandri*, this muscle plays a crucial role in the lateral expansion of the oral cavity. In contrast, the *levator arcus palatini* is less developed in *Lophius*. According to Field (1966), based on the Van Dobben (1935) study, in *L. piscatorius*, this muscle probably acts more as a fixation point. Therefore, the function of promoting the flow of water in the mouth is mainly performed by the *hyoideus* muscle. *L. piscatorius* also differs from *L. alexandri* in the *abductor hyohyoideus* muscle, having four small bands originating in the ceratohyal and passing obliquely outward to insert on the first four branchiostegal rays, in addition to having tendinous fascia medially to *hyohyoideus inferior* that extends to the first branchiostegal ray (Field 1966). In *L. alexandri*, this muscle is limited to a single muscular component restricted to connecting and supporting the first branchiostegal ray.

Another *Lophius*‐shaped fish is *Chaca bankanensis* Bleeker, 1852, a catfish from the Malay Archipelago with similar feeding behavior. The *adductor mandibulae* muscle in this species is roughly triangular (vs. rounded in *L. alexandri*), filling about one‐third of the cheek cavity (vs three‐quarters in *L. alexandri*). *Chaca bankanensis* has a robust and long coronomaxillar cartilage (Diogo et al. 2004b) connected to the maxillary bone, instead of a maxillo‐mandibular ligament as in *L. alexandri*. According to Datovo and Bockmann (2010), the coronomaxillar cartilage was erroneously called *ligamentum primordium* by Diogo et al. (2004b). Other conspicuous differences are the strong *musculus extensor tentaculi* in *Chaca*, with its origin (as a ligament) extending from mesethmoid to the posterior of the frontal bones (vs. origin restricted to lateral ethmoid in *L. alexandri*) and smaller *levator operculi*, with insertion occupying only the dorsal posterior portion of opercle (vs. the entire dorsal portion of opercle).

In *Chaca bankanensis*, the origin of the *levator arcus palatini* is distinguished from *L. alexandri* by its restriction to the sphenotic. In *L. alexandri*, the origin extends from the lateral ethmoid, passing through the entire extension of the frontal until ending in the sphenotic. Another difference in *C. bankanensis* lies in the *protractor hyoideus muscle*, particularly in the *pars ventralis*. This muscle is notable for occupying a large area in the ventral region, as evidenced in the illustrations of Diogo et al. (2004b). The muscle extends to the posterior ceratohyal, contrasting with *L. alexandri*, where the muscle is limited to the anterior ceratohyal.

The lack of *retractor tentaculi* in *L. alexandri* is certainly a homoplasy with *C. bankanensis* because the presence of this muscle is considered a critical structural transformation that occurs in catfishes (Adriaens and Verraes 1997). Therefore, the cephalic musculature of *L. alexandri* is a portrait of its phylogenetic history, besides head morphology and predatory behavior, and reflects the plasticity of musculature arrangements.

The number of studies on comparative anatomy in fish is considerable in the literature. When referring to cephalic musculatures, increased studies in certain groups have revealed valuable information about evolutionary changes that promote a significant understanding of biological diversity and evolutionary adaptations (e.g., Datovo and Bockmann 2010; Datovo and Vari 2013, 2014; Diogo et al. 2003; Diogo 2003; Diogo 2007b; Sarmento‐Soares and Porto 2006). However, many groups still lack data that could clarify long‐standing questions about the phylogenetic differences and how these differences may influence the species' way of life.

## Author Contributions

**Rafael da Silva Marques:** investigation, formal analysis, writing — original draft, data curation. **Isabela Ohara:** investigation, writing — original draft, formal analysis. **Oscar Akio Shibatta:** conceptualization, validation, formal analysis, supervision, writing — original draft, writing — review and editing, funding acquisition, data curation, investigation.

## Peer Review

The peer review history for this article is available at https://www.webofscience.com/api/gateway/wos/peer-review/10.1002/jmor.70056.

## Supporting information

Supplementary material New.

## Data Availability

The data are new and available in the article.
