# Supplementary material for: Cephalic Musculature of the Pacman Catfish Lophiosilurus alexandri Steindachner, 1876 (Siluriformes, Pseudopimelodidae)
Source: J Morphol. 2025 May 24;286(5):e70056. doi: 10.1002/jmor.70056 (PMC12102645; doi:10.1002/jmor.70056)
Supplement: Supplementary file 1 — Supplementary material New. [file JMOR-286-e70056-s001.docx]

**Supplementary material**

**Cephalic musculature of the Pacman catfish *Lophiosilurus alexandri* Steindachner, 1876 (Siluriformes, Pseudopimelodidae)**

Rafael da Silva Marques, Isabela Ohara & Oscar Akio Shibatta

**Table S1.** Pseudopimelodidae myologic character codification. Numerations of musculature characters continue with other morphological characters based on those of Shibatta et al. (2021). See the text for the detailed explanation.

|  | *Pimelodus maculatus* | *Pimelodus microstoma* | *Pseudopimelodus mangurus* | *Lophiosilurus alexandri* | *Lophiosilurus fowleri* | *Batrochoglanis labrosus* |
| --- | --- | --- | --- | --- | --- | --- |
| 64) *Adductor mandibulae*, shape | 0 | 0 | 1 | 1 | 1 | 1 |
| 65) *Adductor mandibulae*, *pars malaris*, tendinous intersection | 0 | 0 | 0 | 1 | 0 | 1 |
| 66) *Adductor mandibulae*, *pars malaris* and *pars rictalis*, lateral view, partial division restricted only to their anterior region | 0 | 0 | 1 | 0 | 0 | 0 |
| 67) *Adductor mandibulae*, *pars stegalis*, *epistegalis*, evidence | 0 | 0 | 1 | 0 | 0 | 0 |
| 68) *Levator arcus palatini*, origin, lateral ethmoid included | 0 | 0 | 1 | 1 | 1 | 1 |
| 69) *Levator arcus palatini*, posterior of origin, sphenotic included | 0 | 0 | 0 | 1 | 1 | 0 |
| 70) *Levator arcus palatini*, insertion, covered by a crest | 0 | 0 | 1 | 0 | 0 | 0 |
| 71) *Levator arcus palatini*, length from origin to insertion | 0 | 0 | 1 | 2 | 2 | 2 |
| 72) *Adductor arcus palatini*, covered by the *levator arcus palatini* | 0 | 0 | 1 | 0 | 0 | 1 |
| 73) *Adductor arcus palatini*, size | 0 | 0 | 1 | 0 | 0 | 1 |
| 74) *Levator operculi*, origin including the *processus opercularis* | 0 | 1 | 1 | 1 | 0 | 0 |
| 75) *Levator operculi* covering the *processus opercularis* | 0 | 1 | 1 | 1 | 1 | 0 |
| 76) *Dilatator operculi*, beginning of origin | 0 | 0 | 0 | 1 | 1 | 0 |
| 77) *Dilatator operculi*, end of origin | 0 | 0 | 0 | 0 | 1 | 0 |
| 78) *Dilatator operculi*, visible in lateral view (covered by the adductor mandibulae) | 0 | 0 | 1 | 0 | 1 | 1 |
| 79) *Adductor operculi*, size | 0 | 0 | 0 | 2 | 1 | 1 |
| 80) *Extensor tentaculi*, *pars medialis*, number of parts | 0 | 0 | 1 | 0 | 0 | 0 |
| 81) *Protractor hyoideus*, *pars lateralis*, origin | 0 | 0 | 0 | 1 | 1 | 0 |
| 82) *Hyohyoideus inferior*, branchiostegal rays covering | 0 | 0 | 1 | 0 | 0 | 0 |
| 83) *Intermandibularis*, shape | 0 | 0 | 0 | 1 | 1 | 1 |
| 84) *Protractor externi mandibularis tentaculi*, origin on the middle of the ceratohyal | 0 | 0 | 0 | 1 | 1 | 1 |

**Table S2.** Data matrix of morphological characters analyzed in the contribution.

*Pimelodus maculatus*

0 ? 0 0 ? 0 0 0 0 1 0 1 1 0 0 1 0 0 0 0 0 0 0 0 0 0 1 0 0 0 0 1 1 0 1 0 0 0 0 1 1 1 0 1 0 0 2 2 0 0 3 0 0 0 1 ? 0 0 2 0 0 0 0 0 0 0 0 0 0 0 0 0 0 0 0 0 0 0 0 0 0 0 0 0

*Pimelodus microstoma*

0 ? 0 0 ? 0 0 0 0 1 0 1 1 0 0 1 0 0 0 0 0 0 0 0 0 0 1 0 0 0 0 1 1 0 1 0 0 0 0 1 1 1 0 1 0 0 ? 2 0 0 3 0 0 0 1 0 0 0 2 0 0 0 0 0 0 0 0 0 0 0 0 0 0 1 1 0 0 0 0 0 0 0 0 0

*Pseudopimelodus mangurus*

1 0 1 0 0 1 0 1 0 0 1 0 1 0 0 0 1 1 1 1 1 1 1 1 0 1 0 1 1 0 1 0 0 0 0 1 0 0 1 1 2 0 1 0 1 1 0 0 0 1 1 1 0 1 1 1 1 0 2 0 0 0 1 1 0 1 1 1 0 1 1 1 1 1 1 0 0 1 0 1 0 1 0 0

*Lophiosilurus alexandri*

1 1 0 0 0 0 0 1 0 0 0 0 1 1 1 0 1 1 1 1 1 0 1 1 1 0 0 1 0 3 0 1 1 0 0 1 1 0 1 0 2 0 0 0 1 1 2 2 1 3 2 1 0 2 1 1 1 1 1 1 0 0 1 1 1 0 0 1 1 0 2 0 0 1 1 1 0 0 2 0 1 0 1 1

*Lophiosilurus fowleri*

1 1 0 0 0 0 0 1 0 0 0 0 1 1 1 0 1 1 1 1 1 0 1 2 1 0 0 1 0 3 0 1 1 0 0 1 1 0 1 0 2 0 1 0 1 1 2 2 1 3 1 1 0 2 1 1 1 1 1 1 0 0 1 1 0 0 0 1 1 0 2 0 0 0 1 1 1 1 1 0 1 0 1 1

*Batrochoglanis labrosus*

1 0 0 1 0 1 2 1 1 1 1 0 1 1 1 0 1 1 1 1 1 0 0 2 1 0 0 2 0 3 0 0 0 1 0 1 0 1 1 0 1 1 1 0 1 1 0 0 0 2 1 1 1 1 1 1 1 1 1 0 1 1 1 1 1 0 0 1 0 0 2 1 1 0 0 0 0 1 1 0 0 0 1 1

Synapomorphies (morphological characters (1 to 63) are based on those of Shibatta et al. (2021); musculature characters (64–84) are from this contribution and highlighted in bold)

*Pimelodus maculatus*:

No autapomorphies

*Pimelodus microstoma*:

No autapomorphies

*Pseudopimelodus mangurus*:

Char. 3: 0 > 1

Char. 22: 0 > 1

Char. 26: 0 > 1

Char. 29: 0 > 1

Char. 31: 0 > 1

**Char. 66: 0 > 1**

**Char. 67: 0 > 1**

**Char. 70: 0 > 1**

**Char. 80: 0 > 1**

**Char. 82: 0 > 1**

*L_alexandri*:

Char. 43: 1 > 0

Char. 51: 1 > 2

**Char. 78: 1 > 0**

**Char. 79: 1 > 2**

*Lophiosilurus fowleri*:

**Char. 77: 0 > 1**

*Batrochoglanis labrosus*:

Char. 4: 0 > 1

Char. 7: 0 > 2

Char. 9: 0 > 1

Char. 28: 1 > 2

Char. 34: 0 > 1

Char. 38: 0 > 1

Char. 53: 0 > 1

Char. 61: 0 > 1

Char. 62: 0 > 1

**Char. 75: 1 > 0**

Node 7 (*Pimelodus microstoma* + Pseudopimelodidae spp.):

No synapomorphies

Node 8 (Pseudopimelodidae spp.):

Char. 1: 0 --> 1

Char. 8: 0 --> 1

Char. 12: 1 --> 0

Char. 16: 1 --> 0

Char. 17: 0 --> 1

Char. 18: 0 --> 1

Char. 19: 0 --> 1

Char. 20: 0 --> 1

Char. 21: 0 --> 1

Char. 27: 1 --> 0

Char. 28: 0 --> 1

Char. 35: 1 --> 0

Char. 36: 0 --> 1

Char. 39: 0 --> 1

Char. 43: 0 --> 1

Char. 44: 1 --> 0

Char. 45: 0 --> 1

Char. 46: 0 --> 1

Char. 51: 3 --> 1

Char. 52: 0 --> 1

Char. 54: 0 --> 1

Char. 57: 0 --> 1

Char. 63: 0 --> 1

**Char. 64: 0 --> 1**

**Char. 68: 0 --> 1**

**Char. 78: 0 --> 1**

Node 9 (*Lophiosilurus alexandri* + *L. fowleri*):

Char. 2: 0 --> 1

Char. 37: 0 --> 1

Char. 49: 0 --> 1

Char. 54: 1 --> 2

Char. 60: 0 --> 1

**Char. 69: 0 --> 1**

**Char. 76: 0 --> 1**

**Char. 81: 0 --> 1**

Node 10 (*Batrochoglanis labrosus* + *Lophiosilurus* spp.):

Char. 14: 0 --> 1

Char. 15: 0 --> 1

Char. 25: 0 --> 1

Char. 30: 0 --> 3

Char. 40: 1 --> 0

Char. 58: 0 --> 1

Char. 59: 2 --> 1

**Char. 79: 0 --> 1**

**Char. 83: 0 --> 1**

**Char. 84: 0 --> 1**

**Table S3.** Consistency Index of characters

|  | 1 | 2 | 3 | 4 | 5 | 6 | 7 | 8 | 9 | 10 |
| --- | --- | --- | --- | --- | --- | --- | --- | --- | --- | --- |
| 0 | 1.00 | 1.00 | 1.00 | 1.00 | 1.00 | 0.50 | 1.00 | 1.00 | 1.00 | 0.50 |
| 10 | 0.50 | 1.00 | 1.00 | 1.00 | 1.00 | 1.00 | 1.00 | 1.00 | 1.00 | 1.00 |
| 20 | 1.00 | 1.00 | 0.50 | 0.67 | 1.00 | 1.00 | 1.00 | 1.00 | 1.00 | 1.00 |
| 30 | 1.00 | 0.50 | 0.50 | 1.00 | 1.00 | 1.00 | 1.00 | 1.00 | 1.00 | 1.00 |
| 40 | 0.50 | 0.50 | 0.50 | 1.00 | 1.00 | 1.00 | 0.50 | 0.50 | 1.00 | 1.00 |
| 50 | 1.00 | 1.00 | 1.00 | 1.00 | 1.00 | 1.00 | 1.00 | 1.00 | 1.00 | 1.00 |
| 60 | 1.00 | 1.00 | 1.00 | 1.00 | 0.50 | 1.00 | 1.00 | 1.00 | 1.00 | 1.00 |
| 70 | 1.00 | 0.50 | 0.50 | 0.33 | 0.50 | 1.00 | 1.00 | 0.50 | 1.00 | 1.00 |
| 80 | 1.00 | 1.00 | 1.00 | 1.00 |  |  |  |  |  |  |

**Table S4.** Homoplasy Index of characters

|  | 1 | 2 | 3 | 4 | 5 | 6 | 7 | 8 | 9 | 10 |
| --- | --- | --- | --- | --- | --- | --- | --- | --- | --- | --- |
| 0 | 0.00 | 0.00 | 0.00 | 0.00 | 0.00 | 0.50 | 0.00 | 0.00 | 0.00 | 0.50 |
| 10 | 0.50 | 0.00 | 0.00 | 0.00 | 0.00 | 0.00 | 0.00 | 0.00 | 0.00 | 0.00 |
| 20 | 0.00 | 0.00 | 0.50 | 0.33 | 0.00 | 0.00 | 0.00 | 0.00 | 0.00 | 0.00 |
| 30 | 0.00 | 0.50 | 0.50 | 0.00 | 0.00 | 0.00 | 0.00 | 0.00 | 0.00 | 0.00 |
| 40 | 0.50 | 0.50 | 0.50 | 0.00 | 0.00 | 0.00 | 0.50 | 0.50 | 0.00 | 0.00 |
| 50 | 0.00 | 0.00 | 0.00 | 0.00 | 0.00 | 0.00 | 0.00 | 0.00 | 0.00 | 0.00 |
| 60 | 0.00 | 0.00 | 0.00 | 0.00 | 0.50 | 0.00 | 0.00 | 0.00 | 0.00 | 0.00 |
| 70 | 0.00 | 0.50 | 0.50 | 0.67 | 0.50 | 0.00 | 0.00 | 0.50 | 0.00 | 0.00 |
| 80 | 0.00 | 0.00 | 0.00 | 0.00 |  |  |  |  |  |  |

**Table S5.** Retention Index of characters

|  | 1 | 2 | 3 | 4 | 5 | 6 | 7 | 8 | 9 | 10 |
| --- | --- | --- | --- | --- | --- | --- | --- | --- | --- | --- |
| 0 | 1.00 | 1.00 | 1.00 | 1.00 | 1.00 | 0.00 | 1.00 | 1.00 | 1.00 | 0.50 |
| 10 | 0.00 | 1.00 | 1.00 | 1.00 | 1.00 | 1.00 | 1.00 | 1.00 | 1.00 | 1.00 |
| 20 | 1.00 | 1.00 | 0.50 | 0.50 | 1.00 | 1.00 | 1.00 | 1.00 | 1.00 | 1.00 |
| 30 | 1.00 | 0.00 | 0.00 | 1.00 | 1.00 | 1.00 | 1.00 | 1.00 | 1.00 | 1.00 |
| 40 | 0.50 | 0.50 | 0.50 | 1.00 | 1.00 | 1.00 | 0.00 | 0.00 | 1.00 | 1.00 |
| 50 | 1.00 | 1.00 | 1.00 | 1.00 | 1.00 | 1.00 | 1.00 | 1.00 | 1.00 | 1.00 |
| 60 | 1.00 | 1.00 | 1.00 | 1.00 | 0.00 | 1.00 | 1.00 | 1.00 | 1.00 | 1.00 |
| 70 | 1.00 | 0.00 | 0.00 | 0.00 | 0.00 | 1.00 | 1.00 | 0.50 | 1.00 | 1.00 |
| 80 | 1.00 | 1.00 | 1.00 | 1.00 |  |  |  |  |  |  |

**Table S6.** Rescaled Consistency Index of characters

|  | 1 | 2 | 3 | 4 | 5 | 6 | 7 | 8 | 9 | 10 |
| --- | --- | --- | --- | --- | --- | --- | --- | --- | --- | --- |
| 0 | 1.00 | 1.00 | 1.00 | 1.00 | 1.00 | 0.00 | 1.00 | 1.00 | 1.00 | 0.25 |
| 10 | 0.00 | 1.00 | 1.00 | 1.00 | 1.00 | 1.00 | 1.00 | 1.00 | 1.00 | 1.00 |
| 20 | 1.00 | 1.00 | 0.25 | 0.3 | 1.00 | 1.00 | 1.00 | 1.00 | 1.00 | 1.00 |
| 30 | 1.00 | 0.00 | 0.00 | 1.00 | 1.00 | 1.00 | 1.00 | 1.00 | 1.00 | 1.00 |
| 40 | 0.25 | 0.25 | 0.25 | 1.00 | 1.00 | 1.00 | 0.00 | 0.00 | 1.00 | 1.00 |
| 50 | 1.00 | 1.00 | 1.00 | 1.00 | 1.00 | 1.00 | 1.00 | 1.00 | 1.00 | 1.00 |
| 60 | 1.00 | 1.00 | 1.00 | 1.00 | 0.00 | 1.00 | 1.00 | 1.00 | 1.00 | 1.00 |
| 70 | 1.00 | 0.00 | 0.00 | 0.00 | 0.00 | 1.00 | 1.00 | 0.25 | 1.00 | 1.00 |
| 80 | 1.00 | 1.00 | 1.00 | 1.00 | ³ | ³ | ³ | ³ | ³ | ³ |

**Table S7.** Fitness of characters (conc 3)

|  | 1 | 2 | 3 | 4 | 5 | 6 | 7 | 8 | 9 | 10 |
| --- | --- | --- | --- | --- | --- | --- | --- | --- | --- | --- |
| 0 | 1.00 | 1.00 | 1.00 | 1.00 | 1.00 | 0.75 | 1.00 | 1.00 | 1.00 | 0.75 |
| 10 | 0.75 | 1.00 | 1.00 | 1.00 | 1.00 | 1.00 | 1.00 | 1.00 | 1.00 | 1.00 |
| 20 | 1.00 | 1.00 | 0.75 | 0.75 | 1.00 | 1.00 | 1.00 | 1.00 | 1.00 | 1.00 |
| 30 | 1.00 | 0.75 | 0.75 | 1.00 | 1.00 | 1.00 | 1.00 | 1.00 | 1.00 | 1.00 |
| 40 | 0.75 | 0.75 | 0.75 | 1.00 | 1.00 | 1.00 | 0.75 | 0.75 | 1.00 | 1.00 |
| 50 | 1.00 | 1.00 | 1.00 | 1.00 | 1.00 | 1.00 | 1.00 | 1.00 | 1.00 | 1.00 |
| 60 | 1.00 | 1.00 | 1.00 | 1.00 | 0.75 | 1.00 | 1.00 | 1.00 | 1.00 | 1.00 |
| 70 | 1.00 | 0.75 | 0.75 | 0.60 | 0.75 | 1.00 | 1.00 | 0.75 | 1.00 | 1.00 |
| 80 | 1.00 | 1.00 | 1.00 | 1.00 |  |  |  |  |  |  |

**Table S8.** Phylogenetic indexes of the tree

| Index | Value |
| --- | --- |
| Consistency Index | 0.82 |
| Homoplasy Index | 0.17 |
| Retention Index | 0.76 |
| Rescaled Consistency Index | 0.62 |
| Average Consistency Index | 0.89 |
| Homoplasy Distribution Index | 0.07 |
| Homoplasy Distribution Ratio | 0.39 |
| Total fit | 56.35 |
